# Distinctive gene expression patterns and imprinting signatures revealed in reciprocal crosses between cattle sub-species

**DOI:** 10.1186/s12864-021-07667-2

**Published:** 2021-06-03

**Authors:** Ruijie Liu, Rick Tearle, Wai Yee Low, Tong Chen, Dana Thomsen, Timothy P. L. Smith, Stefan Hiendleder, John L. Williams

**Affiliations:** 1 Davies Research Centre, School of Animal and Veterinary Sciences, The University of Adelaide, Adelaide, Australia; 2 USMARC, USDA-ARS-US Meat Animal Research Center, Clay Center, NE USA; 3 Robinson Research Institute, The University of Adelaide, Adelaide, Australia; 4 Present address: Dipartimento di Scienze Animali, della Nutrizione e degli Alimenti, Università Cattolica del Sacro Cuore, Piacenza, Italy

**Keywords:** Cattle, Fetal development, Transcriptome

## Abstract

**Background:**

There are two genetically distinct subspecies of cattle, *Bos taurus taurus* and *Bos taurus indicus,* which arose from independent domestication events. The two types of cattle show substantial phenotypic differences, some of which emerge during fetal development and are reflected in birth outcomes, including birth weight. We explored gene expression profiles in the placenta and four fetal tissues at mid-gestation from one taurine (*Bos taurus taurus*; Angus) and one indicine (*Bos taurus indicus*; Brahman) breed and their reciprocal crosses.

**Results:**

In total 120 samples were analysed from a pure taurine breed, an indicine breed and their reciprocal cross fetuses, which identified 6456 differentially expressed genes (DEGs) between the two pure breeds in at least one fetal tissue of which 110 genes were differentially expressed in all five tissues examined. DEGs shared across tissues were enriched for pathways related to immune and stress response functions. Only the liver had a substantial number of DEGs when reciprocal crossed were compared among which 310 DEGs were found to be in common with DEGs identified between purebred livers; these DEGs were significantly enriched for metabolic process GO terms. Analysis of DEGs across purebred and crossbred tissues suggested an additive expression pattern for most genes, where both paternal and maternal alleles contributed to variation in gene expression levels. However, expression of 5% of DEGs in each tissue was consistent with parent of origin effects, with both paternal and maternal dominance effects identified.

**Conclusions:**

These data identify candidate genes potentially driving the tissue-specific differences between these taurine and indicine breeds and provide a biological insight into parental genome effects underlying phenotypic differences in bovine fetal development.

**Supplementary Information:**

The online version contains supplementary material available at 10.1186/s12864-021-07667-2.

## Background

There are substantial phenotypic and genetic differences among cattle breeds, in particular between indicine and taurine breeds (Bovine HapMap Consortium 2009). The taurine and indicine subspecies of cattle arose from independent domestication events resulting in a high degree of genetic divergence [1]. Phenotypically, indicine cattle are more tolerant of hot, humid environments and show greater resistance to parasites such as ticks; hence they are better adapted to survive in tropical areas [2]. However, the productivity of indicine cattle is lower than taurine cattle across a range of traits when measured in temperate zones, including growth and meat quality. Crossbreeding has been used to harness the positive traits of the two types to improve the performance of cattle in tropical environments [3]. Genes such as *MSRB3* and *PLAG1,* which are involved in energy and muscle metabolism, have been shown to have subspecies-specific alleles that affect weight and body condition [4]. However, the genetic factors involved in adaptation to tropical conditions remain largely unknown.

Phenotypic differences between indicine and taurine breeds emerge during fetal development [5] and are reflected in birth outcomes, including birth weight [6]. Fetal growth rate accelerates after mid-gestation (~day 150) [7] and subspecies-specific phenotypes emerge. For example, taurine cattle have a greater myotube cross sectional area and greater bone size than indicine cattle at day 153 [8, 9]. Maternally inherited genes have been shown to contribute disproportionately to myofiber development and muscle and bone in reciprocal crosses, suggesting parent-of-origin imprinting effects [8, 9].

Advances in genome sequencing technology have facilitated the detailed exploration of transcriptome complexity and dynamics. Studies of gene expression in adult bovine tissues, including muscle [10], liver [11, 12], mammary gland [13] and adipose tissue [14] from either taurine or indicine breeds have identified genetic variation associated with differences in feed efficiency, milk composition and deposition of intramuscular fat. However, there is little information available on differences in gene expression between breeds during fetal development. A comparison of gene expression between taurine and indicine breeds may provide biological insights into the origin of their phenotypic differences.

This study investigated the transcriptome of the placenta and four somatic tissues at mid-gestation from two cattle breeds (Angus and Brahman) and their reciprocal crosses. The differentially expressed genes (DEGs) detected between the breeds and between the reciprocal crosses at this fetal stage represent candidates that may be involved in establishing phenotypic differences between the cattle subspecies.

## Results

### Expression profiles of five tissues

A total of 120 samples were analysed, which comprised brain, liver, lung, muscle and placenta samples from 3 pure Angus, 3 pure Brahman, 3 Brahman cross Angus and 3 Angus cross Brahman fetuses. Between 60 and 100 M 100 bp PE reads, or 90-130 M 75 bp PE reads per sample passed quality control. Reads were aligned to the extended Brahman reference genome (UOA_brahman_1 plus non-PAR Y chromosome from UOA_angus_1) using *hisat2* with default settings, giving an average mapping rate of 89%. The total number of expressed genes among samples ranged from 16,368 to 17,013 and showed no substantial variation between tissues. There was a high correlation coefficient between expression of the same genes in each tissue in pure bred Brahman (Bi) and Angus (Bt) (Supplementary Fig. 1a-e). There were 14,143 genes expressed in all tissues (Supplementary Fig. 1f) with 5 genes consistently represented among the 20 most abundant transcripts in all five tissues: Insulin-Like Growth Factor 2 (*IGF2*), Eukaryotic Translation Elongation Factor 1 Alpha 1 (*EEF1A1*), Collagen Type III Alpha 1 Chain (*COL3A1*), Actin Beta (*ACTB*) and the paternally expressed gene 3 (*PEG3*).

**Fig. 1:**
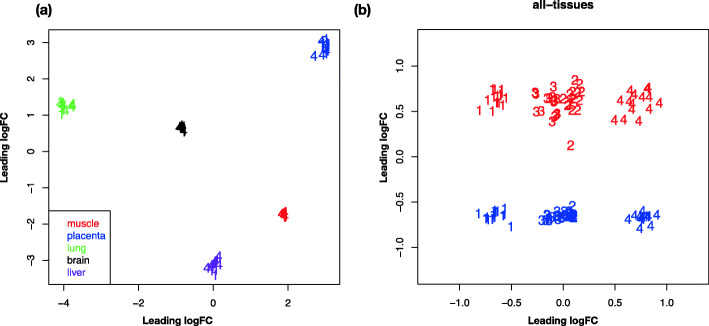
Multi-dimensional scaling (MDS) plot of sample expression profiles in five tissues. **a** The first two dimensions separate the samples by tissue type. **b** After accounting for the tissue source, samples are separated by genetic group in the first dimension (X-axis) and by sex in the second dimension (Y-axis). (1-pure Bt, 2-BtXBi, 3-BiXBt, 4-pure Bi. Male samples are shown in blue and female red)

Multi-scaling analysis grouped samples from each of the 5 tissues into tight clusters which were distinct from each other (Fig. 1a)*.* A multi-factor model was used to account for and remove tissue effects, after which a PCA separated the samples by genetic groups in the first principle component (x-axis) and by sex in the second principle component (y-axis) (Fig. 1b). The expression for each tissue from each genetic type showed the same pattern within sex, with the 2 purebred groups well separated for all tissues, while the reciprocal crosses were less well separated (Supplementary Fig. 2a-e). The 20 most highly expressed genes in each tissue are reported in Supplementary Table 1.

### Differential gene expression between purebred groups

There were 1085, 1495, 1935, 2515 and 2645 genes for which the normalized average number of mapped reads (CPM) differed significantly between purebred Bt and Bi brain, placenta, lung, liver and muscle, respectively. We designated these as differentially expressed genes (DEGs). Muscle had the largest number of DEGs among the tissues studied, but about 84% of these showed a fold change (FC) < 2, while in other tissues ~ 62–72% showed a FC < 2. The most significantly enriched gene ontology (GO) biological process and Kyoto Encyclopedia of Genes and Genomes (KEGG) pathways in muscle included collagen metabolic process (GO:0032963); collagen fibril organization (GO:0030199); amino sugar and nucleotide sugar metabolism (bta00520) and glycine, serine and threonine metabolism (bta00260). Genes in all four of these pathways had higher expression in Bt than in Bi.

Among the DEGs, ~ 10% in each tissue were lncRNAs. About 92% of DE lncRNAs had the opposite transcriptional direction to differentially expressed genes located within 100 kb.

### DEGs common to all five tissues in pure-bred groups

**Fig. 2:**
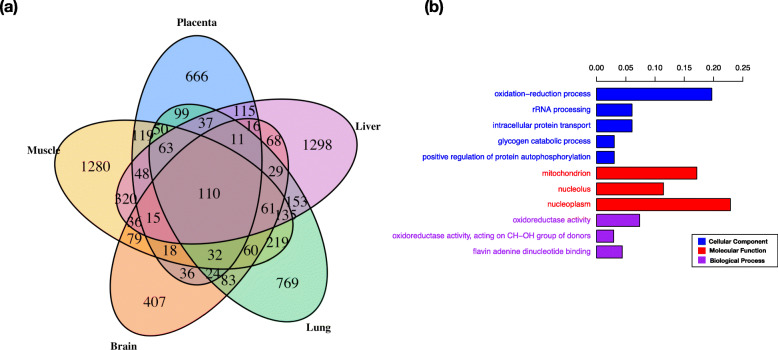
DEG across 5 tissues. **a** Venn diagram depicting the distribution of DEGs across five tissues at FDR cut off 0.05. **b** Significantly enriched gene ontology terms for biological process (purple), Molecular function (red) and cellular component (blue) for 87 annotated DEGs genes that were in common across all five tissues. Bars indicate the percentage of DEGs in the GO term

There were 110 DEGs between Bi and Bt in common for all five tissues, comprising 50 annotated protein-coding genes, 42 genes lacking annotation in the reference genome and 18 lncRNAs (Fig. 2a). Alignment of the unannotated protein-coding genes to known genes in other cattle and ruminant reference genomes facilitated the annotation of 37 of the unnamed DEGs, based on > 90% sequence identity. Of the 87 genes for which annotation was obtained (See Supplementary Table 2) and that were DE in all five tissues between the purebred animals, 84 had consistent relative abundance between subspecies Bt and Bi with respect to genotype in all tissues. The 3 exceptions were Aldehyde Oxidase 1 (*AOX1*), Choline Dehydrogenase (*CHDH*), Syntaxin 11 (*STX11*), whose expression was in a different direction (Bt vs Bi) in the liver compared with the other 4 tissues. GO pathway analysis of the set of 87 annotated genes showed that they were significantly enriched in 10 GO terms with *p*-value < 0.05, including oxidation-reduction process (GO:0055114), intracellular protein transport (GO:0006886), glycogen catabolic process (GO:0005980), positive regulation of protein autophosphorylation (GO:0031954) (Fig. 2b).

### Tissue-specific genes between purebred groups

Genes that were DE between purebred Bt and Bi in only one of the five tissues examined were considered tissue specific DEGs. Using an FDR cut-off of < 0.05 and FC ≥ 2, brain, liver, lung, muscle and placenta had 187, 328, 289, 388 and 191 tissue-specific DEGs respectively. GO biological process pathway enrichment analysis for these filtered tissue-specific DEGs identified 54 GO terms (Supplementary Table 3). The liver-specific DEGs were enriched for 6 GO terms including ion binding (GO:0043167) and primary metabolic processes (GO:0044238). Muscle was enriched for 9 GO terms including the collagen fibril organization pathway (GO:0030199). Brain was also enriched for 9 GO terms that included pathways involved with detection of stimulus (GO:0050906) and nervous system processes (GO:0050087). Lung was enriched for 10 GO terms, most of which were related to fundamental biological processes, including regulation of molecular function (GO:0065009) and cellular response to endogenous stimulus (GO:0071495). Placenta was enriched for 20 GO terms which were linked to proton-transporting V-type ATPase (GO:0033176) and domain small molecule metabolic process (GO 0044281).

### Differential gene expression between crossbred groups

Comparison of transcript abundance between the reciprocal cross-bred groups (Bt x Bi and Bi x Bt) did not reveal a substantial number of DEGs (< 20/tissue at FDR < 0.05), except for liver which had 2473 DEGs. However, only 143 (5.8%) of the liver DEGs had a fold change greater than 2. We performed GO biological process pathway enrichment analysis and KEGG pathway enrichment analysis for the protein coding DE genes with > 2-fold change. The GO analysis showed that DEGs were significantly enriched in 6 GO terms, including: macromolecule metabolic process (GO:0043170), primary metabolic process (GO:0044238), cellular metabolic process (GO:0044237), metabolic process (GO:0008152), nitrogen compound metabolic process (GO:0006807) and organic substance metabolic process (GO:0071704) which are all involved in metabolic processes. The only significantly enriched KEGG pathway was metabolic pathways (path: bta01100).

Pairwise comparisons of the DEGs in liver for the 4 genetic groups were performed to explore relationships in expression patterns between pure bred and crossbred concepti. The sire dominated the liver expression pattern in Bt-sired crossbred (Bt x Bi) liver which had 1276 DEGs when compared to purebred Bi liver, versus 219 DEG when compared with purebred Bt liver. However, the dam breed appears to dominate expression pattern in Bi-sired crossbreds, with 317 DEGs in the Bi x Bt crossbred compared with purebred Bt, but 150 DEGs when compared with purebred Bi liver transcripts.

### Expression pattern of DEGs from the purebred groups in comparison with crossbred groups

**Fig. 3:**
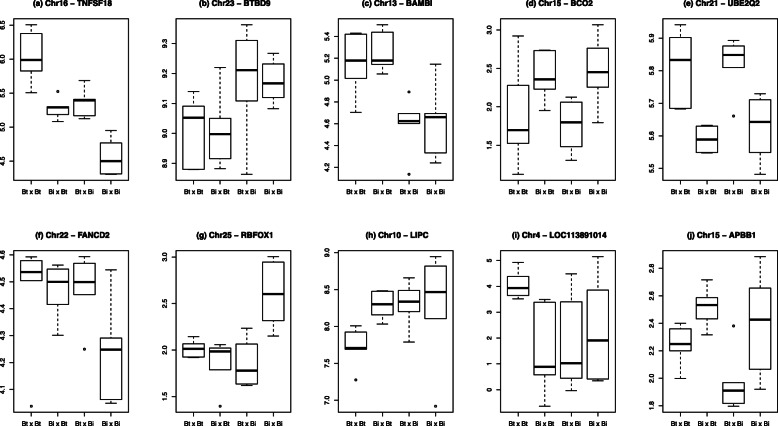
Examples of expression patterns among genotype groups. Boxplots illustrating the different expression patterns observed among the 4 genetics groups: Bt x Bt, Bi x Bt, Bt x Bi and Bi x Bi (sire breed given first). Y-axis is expression level (counts per million) on a log_2_ scale. **a** Taurus driven additive expression, irrespective of parent. **b** Maternal genome driven indicine dominance. **c** Maternal genome driven taurine dominance. **d** Paternal genome driven indicine dominance. **e** Paternal genome driven taurine dominance. **f** Taurine dominant – activation. **g** Taurine dominant - inhibition. **h** Indicine dominant - activation. **i** Indicine dominant – inhibition. **j** complex inheritance

The expression pattern of the 6456 DEGs between tissues of purebred animals was examined in the reciprocal crossbred groups. Of these DEGs 5784 (~ 90%) showed an additive expression pattern where both paternal and maternal genomes contributed to the gene expression levels in the crossbred groups (Fig. 3a), as suggested by the transcript abundance falling approximately midway between that of the two purebred classes. However, transcript abundance of some DEGs (672) was more consistent with parent-of-origin driven expression (Fig. 3b-i). Different types of such effects were observed, predominantly maternal/paternal dominance and Bt or Bi allele derived dominance. The abundance of DEGs between crossbred groups fell into three general categories: co-dominant, dominant and recessive expression patterns, with dominance in some cases driven by either the male or the female (Fig. 3). The number of genes falling into each category are given in Table 1.

**Table 1:** Number of genes showing a parent of origin effect on expression patterns in five tissues

	Brain	Liver	Lung	Muscle	Placenta
Maternal genome driven - Taurine	0	12	2	16	0
Maternal genome driven - Indicine	0	11	4	11	5
Paternal genome driven – Taurine	6	24	9	16	7
Paternal genome driven - Indicine	5	36	13	13	12
Taurine dominant – activation	27	65	40	33	25
Taurine dominant – inhibition	7	30	24	10	12
Indicine dominant – activation	13	38	17	15	10
Indicine dominant - inhibition	2	22	3	15	5

GO analysis of the DEGs that overlapped between tissues showed that they were significantly enriched in 19 GO terms including positive regulation of cellular metabolic process (GO:0009893), positive regulation of nitrogen compound metabolic process (GO:0051173) and membrane-enclosed lumen (GO:0031974). The transcript levels of the DEGs involved in these significantly enriched pathways had exclusively higher expression in the purebred Bt compared with the purebred Bi.

## Discussion

The study of gene expression in prenatal development will help to understand the regulation of fetal tissue-specific growth and development. Our hypothesis was that phenotypic differences between subspecies of cattle may be due, in part, to differential gene expression during mid-gestation. Consistent with this hypothesis, in this study we observed substantial differences in expression between breeds of cattle from the two genetically distinct sub-species *Bos taurus taurus* (Angus) and *Bos taurus indicus* (Brahman). In addition, we observed differential expression of genes in reciprocal crosses between these subspecies, some of which revealed parent-of-origin and breed-of-origin effects on gene expression in five tissues at mid-gestation.

We found that five genes had high levels of expression in all five tissues at this developmental stage (*IGF2*, *EEF1A1*, *COL3A1*, *ACTB* and *PEG3*). These genes play a crucial role in embryonic development and fetal tissue growth, as shown by loss-of-function mutations which result in developmental delay and several diseases including intellectual disability, immune system abnormalities, cerebral abnormalities and abnormally large abdominal organs [15–19]. *EEF1A1* is a member of the eukaryotic elongation factor family that regulates protein synthesis, that is expressed in brain, placenta, lung, liver, kidney, and pancreas in human adults [20]. *COL3A1* is expressed in extensible connective tissues, such as skin and lung. A mutation in *COL3A1* has been linked to vascular disease [21]. Expression levels of *IGF2* have been linked to increased muscle mass [22] and fetal growth [23].

Other highly abundant transcripts showed tissue-specific expression levels which were related to tissue function. Alpha-Fetoprotein (*AFP*) had liver-specific expression and encodes a major plasma protein produced by the liver during fetal development [24]. Two genes that were highly expressed in the muscle were the muscle structural protein genes Myosin Heavy Chain 3 (*MYH3*) and Myosin Binding Protein C, Slow Type (*MYBPC1*) [25, 26]. Genes that play an important role in neurodevelopment including Adenylate Cyclase 1 (*ADCY1*), Stathmin 2 (*STMN2*) and Tubulin Beta 3 Class III (*TUBB3*) were highly expressed and specific to the brain [27–29]. All of these genes had high levels of expression in both the pure breed concepti and the crosses. The lung was the only tissue that did not have any highly expressed tissue-specific genes (cut off Log_2_CPM > 10) at this developmental stage.

Muscle composition and quality of taurine and indicine cattle breeds differs [30] and is largely determined during fetal development [31]. We have previously reported greater fast myotube cross sectional area and greater bone size in taurine than indicine cattle fetuses at day 153 [8, 9]. In the present study we show that muscle contained the highest number of DE genes between purebreds amongst all studied tissues. Significantly enriched pathways included collagen metabolic process, collagen fibril organization, amino sugar and nucleotide sugar metabolism and glycine, serine and threonine metabolism. Genes in all four of these pathways had higher expression in Bt than in Bi fetuses. Although we did not examine gene expression in bone tissue, it is known that fetal muscle and bone growth are linked and collagen pathways also play a major role in bone growth [32].

Intrauterine stress increases the risk of adult disease through fetal programming mechanisms. Increased oxidative stress during embryonic and fetal growth can be caused by environmental and physiological conditions [33], and may affect key transcription factors that can alter gene expression during development [34]. From the GO pathway analysis in the current study, oxidation-reduction processes and oxidoreductase activity were found to be significantly associated with the DEGs between the two pure breeds that were in common to all five tissues.

Heat shock leads to oxidative stress, which has been associated with reduced production performance in *Bos taurus indicus* [35]. During heat stress the steady-state concentration of free radicals is disturbed, resulting in both cellular and mitochondrial oxidative damage [36]. A study of the effects of oxidative stress on cattle fertility indicated that in tropical areas, *Bos taurus taurus* bulls have a higher level of reactive oxygen species (ROS) in their semen than *Bos taurus indicus* bulls [37]. It has been suggested that these high levels of ROS cause major sperm defects in heat stressed *Bos taurus taurus* bulls [34]. In our study, *TXNRD2,* a nuclear genome encoded mitochondrial protein that scavenges reactive oxygen species, had a higher level of expression in Bi than Bt in all tissues. It is possible that *TXNRD2* mediated protection of mitochondrial function may help indicine cattle to better adapt to hot environments.

The *HSD11B1L* encoded protein catalyses the interconversion of inactive to active glucocorticoids, e.g. the conversion of inactive cortisone to the active forms: corticosterone and cortisol. These are key hormones that regulate a variety of physiologic responses to stress through the hypothalamus-pituitary-adrenal (HPA) axis that is responsible for the adaptation of stress responses to restore homeostasis [38]. Higher levels of *HSD11B1L* transcripts were found in all Bi tissues compared with Bt, which may allow indicus cattle to respond more rapidly than taurine cattle to stressful situations, including environmental and biological challenges.

Most of the genes that were DE in all five tissues showed changes in the level of expression in the same direction for all tissues. There were 3 exceptions with different directions of expression in the liver compared with the other 4 tissues. The liver plays an important role in metabolic processes and in immune system function, which affects the response to many diseases [39, 40]. We found that the expression of *AOX1* was higher in all Bi tissues except liver, where it was lower. *AOX1* produces hydrogen peroxide and catalyses the formation of superoxide. Levels of *AOX1* increase in mouse liver following infection [41] suggesting a role in immune response by stimulating host immunity, inflammation and coagulation. Indicine cattle are generally less susceptible to disease than taurine cattle [42, 43]. For example, they are more resistant to ticks [44] and tuberculosis [45]. Interestingly *AOX1* had lower levels of expression in Bi than Bt in tissues other than liver. The significance of this is unclear. The GO terms including genes that were DE between purebreds in this study showed that those involved in metabolic processes generally had significantly higher expression in Bt compared with Bi. Low metabolic rate has been associated with thermotolerance of *Bos taurus indicus* [46].

Interestingly, the genes that were DE between the liver of the pure-bred concepti, that were also differentially expressed between the reciprocal crossbred concepti, showed a higher expression when the sire was taurine for both sexes. For example, a critical nuclear receptor *NR4A1* had a higher level of expression in pure Bt and in the crossbred concepti when the sire was Bt. *NR4A1* is involved in inflammation, apoptosis, and glucose metabolism and also regulates a paternally imprinted gene, *SNRPN,* which affects neurological and spine development [47]. NR4A1 regulates energetic competence of mitochondria and promotes neuronal plasticity. However, studies in animal models and of neuropathologies in humans have shown that sustained expression of this gene results in increased sensitivity to chronic stress [48]. Higher levels of expression in Bt may be related to a reduced tolerance of stress including heat and drought conditions.

Genomic imprinting, which is reflected in a biased level of expression of one autosomal copy of a gene and is dependent on the parent of origin, has been reported in all mammalian species in which it has been assessed, e.g. mice [49], humans [50], and domesticated animals [51]. Both insulin-like growth factor (*IGF2*) and paternally expressed gene 3 (*PEG3*) are imprinted in humans, mice, cattle and other species [52, 53] and the paternally inherited copy is expressed during fetal development, with expression declining rapidly after birth [54]. Both genes play an important role in controlling fetal growth rate and nurturing behaviours in mammals. In the present study, *IGF2* and *PEG3* were highly expressed in all samples across the 4 pure and crossbred groups in all five tissues, suggesting that both *PEG3* and *IGF2* functions are essential at mid-gestation. The overall levels of *PEG3* and *IGF2* transcripts did not differ between breeds or the direction of the cross, although we were unable to assign transcripts to a parent of origin to test for imprinting.

## Conclusion

This study identified a large number of genes that showed significant tissue-specific expression differences between the taurine and the indicine breeds studied. These genes were found to participate in pathways related to tissue-specific function. Genes that were differentially expressed between Angus and Brahman in all tissues were found to relate to functions such as immune response and stress response, that may to some extent explain the higher resilience of Bi cattle. This study also identified genes that putatively have parent or breed of origin-controlled expression patterns. Exploring these further would require e.g. long read Iso-seq data to resolve haplotype specific expression. The current data provide a basis for future research on parental genome effects underlying phenotypic differences in cattle fetal development. Taking these factors into account in breeding and management may improve the welfare and productivity of cross-bred cattle in tropical environments.

## Material and methods

### Animals and sample collection

All animal experiments and procedures described in this study were compliant with national guidelines and approved by the University of Adelaide Animal Ethics Committee which follows ARRIVE Guidelines (https://arriveguidelines.org/) for approval and monitoring all studies involving live animals (Approval No. S-094-2005). The animals and semen used were pure bred Angus (*Bos taurus taurus*) and Brahman (*Bos taurus indicus*) cattle, subsequently referred to as Bt and Bi respectively. Purebred Bt and Bi females (heifers) of approximately 16–20 months of age were maintained on pasture supplemented with silage. The heifers were inseminated with semen of purebred Bt or Bi sires and pregnancy tested by ultrasound scanning. Pregnant heifers and their concepti were humanely sacrificed at day 153 +/− 1 of gestation and the conceptus dissected. Tissues were snap-frozen in liquid nitrogen and then stored at -80 °C as previously described [8]. The five tissues used in this study, brain, liver, lung, muscle and placenta, were taken from 3 male and 3 female concepti, from each of the 4 genetic combinations (Bt x Bt, Bi x Bt, Bt x Bi, Bi x Bi; paternal genome listed first), giving a total of 24 samples per tissue.

### RNA isolation, library preparation and sequencing

Total RNA was isolated from tissues using the RiboZero Gold kit, in accordance with the manufacturer’s recommendations (Illumina, San Diego, CA). Sequencing libraries were prepared with a KAPA Stranded RNA-Seq Library Preparation Kit following the Illumina paired-end library preparation protocol (Illumina, San Diego, CA). Paired-end (PE) sequence reads were produced on an Illumina NextSeq500 platform, 2 × 75 bp for placenta, lung and brain and 2x100bp for liver and muscle.

### Data analysis

FastQC [55] was used to assess read quality and adaptor sequences were removed using cutadapt (Martin, 2011). The UOA_Brahman and UOA_Angus genome assemblies (GCA_003369695.2; GCA_003369685.2) are more contiguous that the ARS-UCD1.2 assembly and are completely phased, for this reason, and that data were produced from Brahman and Angus fetuses, these sequences were chosen as the reference. RNA seq reads were aligned with both UOA_Brahman and UOA_Angus assemblies and better alignment was found using UOA_Brahman. Approximately 93.1% sequences aligned to the Brahman genome whereas only 90.3% sequences aligned to the Angus genome. Therefore, an extended bovine Brahman reference genome, consisting of the autosomes and X chromosome from UOA_Brahman_1 and the non-PAR Y chromosome from UOA_Angus_1 was used in the analyses. Reads were aligned to this reference using hisat2 [56]. The number of annotated clean reads for each gene was calculated using feature counts from the Rsubread package [57] with gene definitions from Refseq and Ensembl annotation v97. Genes with a count per million (CPM) reads below 0.5 were excluded. Multi-dimensional scaling (MDS) plots were created using plotMDS from the *limma* R package. The expression of genes was normalised across the libraries by the Trimmed Mean of M-values (TMM) [58], and potential batch effects due to samples being sequenced in different sequencing runs were accounted for using the RemoveBatchEffect function in the limma package*.* Ignoring sex difference, differentially expressed genes (DEGs) with a false discovery rate (FDR) < 0.05 after down-weighting high variation replicates, were identified using the *limma-voom* R package [59, 60]. Sequences identified as protein-coding genes in the assembly, but lacking names, were annotated using BLASTN with the nucleotide collection nr/nt [61], selecting the top annotated gene if it had more than 90% identity with the unknown gene.

### Functional analysis of DEGs

To facilitate functional analysis of DEGs, cattle gene IDs were converted to homologous human Ensembl gene IDs using BioMart R packages [62]. Kyoto Encyclopedia of Genes and Genomes (KEGG) pathway and Gene Ontology (GO) enrichment analyses of DEGs were performed using the *limma* R package [63]. GO terms for molecular functions (MF), biological processes (BP) and cellular components (CC) were interrogated. Fisher’s exact tests were carried out and an adjusted *P* value calculated using the Benjamini-Hochberg procedure for multiple tests (FDR). GO and KEGG terms with an adjusted P value < 0.05 were considered to be significantly enriched pathways. GSEA software was used to define and plot the pathway networks for DEGs**.**

### Identification of Brahman and Angus gene expression pattern in crossbred groups

Some phenotypes of the concepti differed depending on which was the paternal breed in Bt and Bi reciprocal crosses, e.g. birth weight and mature size [8, 9]. We tested whether this difference was reflected in gene expression differences during fetal development. Genes where total abundance of transcripts was dominated by paternal breed were defined as paternally-driven, or when abundance was dominated by the maternal breed to be maternally-driven. To identify such genes, we used the difference in expression between purebred concepti to define breed-specific transcript abundance. Specifically, the absolute difference of transcript abundance in Bt versus Bi provided a “normalization” of expected abundance for a crossbred conceptus. Using that difference as denominator, we then used the absolute difference in abundance between the reciprocal crosses to identify genes influenced by the parental origin.

For example, if we assume a transcript has a difference in abundance between Angus and Brahman (Bt x Bt / Bi x Bi) of 3.0 (i.e. higher in Angus) and the difference in abundance in Angus-sired crossbreds compared with Brahman-sired crossbreds (Bt x Bi / Bi x Bt) is 2.7, the ratio of differences is 0.9 and is consistent with parental-driven expression. We use an arbitrary threshold of > 0.8 to designate parental-driven expression. We then use the differences in expression between the purebreds and crossbreds to determine if the gene is maternally or paternally driven. For convenience, we define the four genetic groups, i.e., the purebreds and crossbreds, by digits, with Bt xBt, Bi x Bt, Bt x Bi, and Bi x Bi labelled as 1, 2, 3 and 4 respectively. All combinations of pairs of groups were compared, and for each gene the above ratio was calculated. Six combinations of expression differences were obtained: diff1–2, diff1–3, diff1–4, diff2–3, diff2–4 and diff3–4. The threshold was adjusted to identify genes in the following patterns:

1. Maternally driven-taurine: diff2–3/diff1–4 > 0.8, diff1–2/diff1–4 < 0.2, diff3–4/diff1–4 < 0.2, high expression level in Bt
2. Maternally driven-indicine: diff2–3/diff1–4 > 0.8, diff1–2/diff1–4 < 0.2, diff3–4/diff1–4 < 0.2, high expression level in Bi
3. Paternally driven-taurine: diff2–3/diff1–4 > 0.8, diff1–3/diff1–4 < 0.2, diff2–4/diff1–4 < 0.2, high expression level in Bt
4. Paternally driven-indicine: diff2–3/diff1–4 > 0.8, diff1–3/diff1–4 < 0.2, diff2–4/diff1–4 < 0.2, high expression level in Bi
5. Taurine dominant-Inhibition: diff2–3/diff1--4 < 0.2, diff24/ diff1–4 < 0.8, diff3–4/diff1–4 < 0.8, high expression level in Bi
6. Taurine dominant-Activation: diff2–3/diff1–4 < 0.2, diff2–4/ diff1–4 < 0.8, diff3–4/diff1–4 < 0.8, high expression level in Bt
7. Indicine dominant-Inhibition: diff2–3/diff1–4 < 0.2, diff1–2/diff1–4 < 0.8, diff1–3/diff1–4 < 0.8, high expression level in Bt
8. Indicine dominant-Inhibition: diff2–3/diff1–4 < 0.2, diff1–2/diff1–4 < 0.8, diff1–3/diff1–4 < 0.8, high expression level in Bi

Demonstrations of Bi and Bt gene expression patterns in crossbred groups are shown in Supplementary Fig. 3.

## Supplementary Information

**Additional file 1.**

**Additional file 2.**

## Data Availability

Sequence data is available from the GEO under accession number GSE148909.

## Abbreviations

Bi: *Bos taurus taurus*
Bt: *Bos taurus indicus*
DEG: differentially expressed genes
PE reads: paired end reads
MDS: multi-dimensional scaling
PCA: principal components analysis.
CPM: counts per million.
