# Supplementary material for: Distinctive gene expression patterns and imprinting signatures revealed in reciprocal crosses between cattle sub-species
Source: BMC Genomics. 2021 Jun 3;22:410. doi: 10.1186/s12864-021-07667-2 (PMC8176687; doi:10.1186/s12864-021-07667-2)
Supplement: Supplementary file 1 — Additional file 1. [file 12864_2021_7667_MOESM1_ESM.docx]

**Distinctive gene expression patterns and imprinting signatures revealed in reciprocal crosses between cattle sub-species.**

Ruijie Liu, Rick Tearle, Wai Yee Low, Tong Chen, Dana Thomsen, Timothy P.L. Smith, Stefan Hiendleder, John L. Williams^*^

**SUPPLEMENTARY FIGURES**


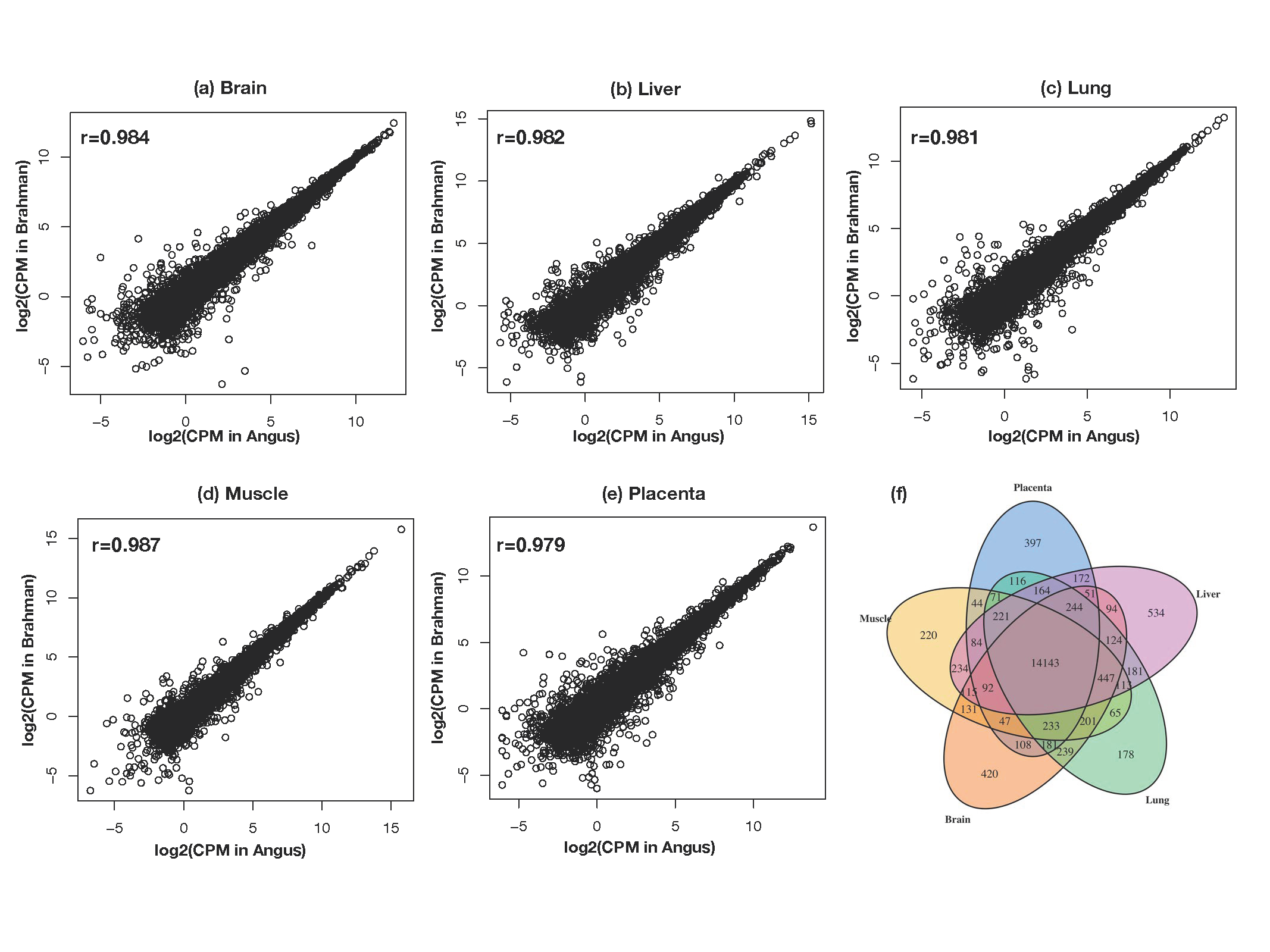


Supplementary Figure 1: Comparison of gene expression levels in five tissues for pure Bt and Bi. (a)-(e) The X and Y axes plot the gene expression counts (log2 count per million) in Bt vs Bi in Brain, Liver, Lung, Muscle and Placenta, respectively. f) Venn diagram shows the overlap of expressed genes in five tissues.

Supplementary Figure 2: Multi-dimensional scaling plots of genetic-difference in each tissue. Male samples are in blue and female samples are in red. The X and Y axes are in log2 fold changes. a) Brain; b) Liver; c) Lung; d) Muscle; e) Placenta.

Supplementary Figure 3: Demonstration of Bi and Bt gene expression pattern in crossbred groups. Only genes differentially expressed between purebred groups are considered. Average expression values from 4 genetic types are obtained. Bt xBt, Bi x Bt, Bt x Bi, and Bi x Bi (paternal genome listed first) are labelled as 1, 2, 3 and 4 respectively.a) Maternal genome driven – Taurine dominance. b) Maternal genome driven – Indicine dominance. c) Paternal genome driven – Taurine dominance. d) Paternal genome driven – Indicine dominance. e) Taurus driven additive expression, irrespective of parent. f) Indicus driven additive expression, irrespective of parent. g) Taurine dominant – inhibition. h) Taurine dominant – activation. i) Indicine dominant – inhibition. j) Indicine dominant – activation.
