# Supplementary material for: Distinctive gene expression patterns and imprinting signatures revealed in reciprocal crosses between cattle sub-species
Source: BMC Genomics. 2021 Jun 3;22:410. doi: 10.1186/s12864-021-07667-2 (PMC8176687; doi:10.1186/s12864-021-07667-2)
Supplement: Supplementary file 2 — Additional file 2. [file 12864_2021_7667_MOESM2_ESM.docx]

**Distinctive gene expression patterns and imprinting signatures revealed in reciprocal crosses between cattle sub-species.**

Ruijie Liu, Rick Tearle, Wai Yee Low, Tong Chen, Dana Thomsen, Timothy P.L. Smith, Stefan Hiendleder, John L. Williams^*^

**SUPPLEMETARY TABLES**

Supplementary Table 1: Top 20 highly expressed genes in five tissues.

| Top 20 highly expressed genes in placenta | | |  |  |  |
| --- | --- | --- | --- | --- | --- |
| Chr | Start | End | Stand | Symbol | average expression (log2CPM) |
| 29 | 13504876 | 13514381 | + | LOC113886273 | 13.6998036 |
| 29 | 12504822 | 12514216 | + | LOC113885740 | 12.2413319 |
| 23 | 36300765 | 36313454 | + | LOC113881926 | 12.1696352 |
| 18 | 1704731 | 1728155 | + | PEG3 | 12.1487294 |
| 15 | 61655547 | 61667673 | + | LOC113905846 | 12.1423213 |
| 13 | 9247781 | 9254044 | + | LOC113902973 | 11.9548201 |
| 23 | 51784919 | 51794891 | + | LOC113881729 | 11.7617333 |
| X | 84976087 | 85002158 | + | CAPN6 | 11.7016996 |
| 25 | 3736097 | 3739524 | - | ACTB | 11.6690322 |
| 29 | 1746722 | 1774493 | - | IGF2 | 11.6635825 |
| 6 | 84881046 | 84938250 | - | SULT1E1 | 11.6152341 |
| 19 | 52342792 | 52345664 | + | ACTG1 | 11.583779 |
| 11 | 49742778 | 49743850 | - | TMSB10 | 11.5490634 |
| 4 | 21652784 | 21669638 | + | LOC113891189 | 11.5204069 |
| 29 | 21999597 | 22007274 | - | LOC113886272 | 11.439267 |
| 27 | 13989220 | 14142127 | + | WWC2 | 11.3191304 |
| 10 | 79581547 | 79590358 | - | NPC2 | 11.2134152 |
| 23 | 35777885 | 35790031 | - | LOC113881912 | 11.2095006 |
| 29 | 11731065 | 11740940 | + | LOC113886288 | 11.2081314 |
| 29 | 12860244 | 12875509 | + | LOC113886290 | 11.1398374 |
| Top 20 highly expressed genes in brain | | |  |  |  |
| Chr | Start | End | Stand | Symbol | average expression (log2CPM) |
| 21 | 65568371 | 65603630 | + | LOC113879939 | 12.3139452 |
| 5 | 89638440 | 89642890 | - | TUBA1A | 11.899499 |
| 10 | 101904614 | 101915069 | + | CALM1 | 11.8604035 |
| 14 | 43120345 | 43178570 | + | STMN2 | 11.6336261 |
| 20 | 9245810 | 9339037 | - | MAP1B | 11.6269479 |
| 19 | 52342792 | 52345664 | + | ACTG1 | 11.3120653 |
| 2 | 126790948 | 126821568 | + | STMN1 | 11.2717125 |
| 11 | 98240094 | 98298529 | + | SPTAN1 | 11.1227417 |
| 8 | 73721987 | 73840519 | + | DPYSL2 | 11.0621011 |
| 7 | 52092400 | 52209325 | + | DPYSL3 | 11.0594361 |
| 4 | 107686367 | 107699081 | - | PEG10 | 10.9799059 |
| 23 | 29182886 | 29187332 | - | TUBB | 10.9425705 |
| 2 | 97178044 | 97475943 | + | MAP2 | 10.9356893 |
| 25 | 8149068 | 8173718 | + | YWHAG | 10.9041657 |
| 18 | 51083202 | 51091539 | - | TUBB3 | 10.8777419 |
| 19 | 46939392 | 47063344 | + | MAPT | 10.8226901 |
| 4 | 43464704 | 43571682 | + | ADCY1 | 10.7398511 |
| 7 | 90600521 | 90609410 | - | EEF2 | 10.7254946 |
| 13 | 29265820 | 29273580 | - | EEF1A2 | 10.6955452 |
| 16 | 78765060 | 78809629 | - | KIF21B | 10.6365828 |
| Top 20 highly expressed genes in liver | | |  |  |  |
| Chr | Start | End | Stand | Symbol | average expression (log2CPM) |
| 1 | 80335480 | 80342557 | - | AHSG | 14.9280801 |
| 6 | 88039295 | 88057657 | + | ALB | 14.8994058 |
| 21 | 58943373 | 58955269 | - | SERPINA1 | 13.9120497 |
| 6 | 88065424 | 88087062 | + | AFP | 13.4403382 |
| 1 | 134958638 | 135044784 | - | LOC113895043 | 13.2374078 |
| 15 | 35604141 | 35605767 | - | LOC113905582 | 12.6025102 |
| 17 | 69741253 | 69749107 | + | FGB | 12.2571716 |
| X | 128075530 | 128539068 | + | GPC3 | 11.9754052 |
| 11 | 77137851 | 77179834 | + | APOB | 11.7053917 |
| 13 | 67579296 | 67611649 | + | ITIH2 | 11.618076 |
| 4 | 107686367 | 107699081 | - | PEG10 | 11.5926797 |
| 6 | 86528024 | 86580214 | - | GC | 11.5455457 |
| 17 | 69774759 | 69782875 | - | FGA | 11.3468167 |
| 18 | 1704731 | 1728155 | + | PEG3 | 11.1051372 |
| 1 | 80284653 | 80299937 | - | FETUB | 10.9786776 |
| 17 | 69794842 | 69802922 | - | FGG | 10.9332044 |
| 22 | 12700318 | 12714256 | + | ITIH3 | 10.7482299 |
| 3 | 8319332 | 8320657 | + | APOA2 | 10.7206371 |
| 8 | 103307653 | 103320100 | - | AMBP | 10.6925629 |
| 2 | 63594578 | 64814251 | + | NCKAP5 | 10.6521329 |
| Top 20 highly expressed genes in lung | | |  |  |  |
| Chr | Start | End | Stand | Symbol | average expression (log2CPM) |
| 29 | 1746722 | 1774493 | - | IGF2 | 13.2042136 |
| 2 | 7134607 | 7174433 | - | COL3A1 | 12.7579585 |
| 21 | 65568371 | 65603630 | + | LOC113879939 | 12.2590567 |
| 9 | 13105107 | 13111604 | - | EEF1A1 | 11.9331407 |
| 13 | 25895716 | 25963127 | + | LOC113903214 | 11.8854638 |
| 19 | 37532052 | 37550039 | + | COL1A1 | 11.6442336 |
| 3 | 116885591 | 116976466 | - | COL6A3 | 11.6423404 |
| 3 | 106719811 | 107060340 | - | MACF1 | 11.4133616 |
| 14 | 77275253 | 77285338 | - | CA3 | 11.0632242 |
| 2 | 103263988 | 103333284 | - | FN1 | 11.0124467 |
| 7 | 90600521 | 90609410 | - | EEF2 | 11.0106332 |
| 11 | 36862372 | 37073566 | + | SPTBN1 | 10.8841484 |
| 23 | 18804260 | 18809940 | + | HSP90AB1 | 10.8654912 |
| 12 | 84456017 | 84589330 | - | COL4A1 | 10.8101364 |
| 22 | 48136990 | 48150444 | - | RPSA | 10.7914474 |
| 29 | 10206701 | 10299311 | + | AHNAK | 10.7449534 |
| 25 | 40234488 | 40252770 | + | SRRM2 | 10.7309843 |
| 7 | 47906558 | 47929571 | + | SPARC | 10.7219419 |
| 25 | 41018318 | 41020575 | + | RPS2 | 10.7182363 |
| 10 | 7100359 | 7105068 | - | RPL4 | 10.6901173 |
| Top 20 highly expressed genes in muscle | | |  |  |  |
| Chr | Start | End | Stand | Symbol | average expression (log2CPM) |
| 2 | 17900389 | 18176615 | + | TTN | 15.7263787 |
| X | 30451046 | 33107990 | - | DMD | 13.44033 |
| 19 | 30444588 | 30474852 | - | LOC113877399 | 12.9929557 |
| 2 | 7134607 | 7174433 | - | COL3A1 | 12.7530116 |
| 2 | 44319518 | 44538758 | + | NEB | 12.5479877 |
| 29 | 38930699 | 41249691 | - | DLG2 | 12.1956708 |
| 19 | 30643315 | 30664308 | - | MYH3 | 12.1536394 |
| 5 | 54748044 | 54849459 | - | MYBPC1 | 12.1404033 |
| 21 | 65568371 | 65603630 | + | LOC113879939 | 11.7040716 |
| 6 | 50128676 | 50604276 | + | PCDH7 | 11.4095231 |
| 10 | 42742821 | 43553521 | + | RORA | 11.3070367 |
| X | 92746655 | 92875008 | + | NRK | 11.2037122 |
| 28 | 22239873 | 24150230 | - | CTNNA3 | 11.2001939 |
| 19 | 37532052 | 37550039 | + | COL1A1 | 11.1746899 |
| 25 | 11541914 | 12757716 | + | AUTS2 | 11.1347041 |
| 12 | 23965480 | 24000775 | + | POSTN | 11.1327618 |
| 22 | 56132397 | 56935219 | - | RBMS3 | 10.9463302 |
| 29 | 1746722 | 1774493 | - | IGF2 | 10.8777777 |
| 8 | 29599753 | 29857371 | + | NFIB | 10.8285828 |
| 3 | 84324798 | 84725574 | - | NFIA | 10.7780565 |

Supplementary Table 2: Genes with annotation that are differentially expressed between Bi and Bt in all 5 tissues.

| Entrez ID | Chromosome | Start | End | Strand | Gene ID | Symbol | gene_type |
| --- | --- | --- | --- | --- | --- | --- | --- |
| 1266359 | 22 | 17781576 | 17793598 | + | gene24995 | RPP14 | protein_coding |
| 541330 | 8 | 38694509 | 38752815 | + | gene10203 | ERMP1 | protein_coding |
| 478272 | 7 | 89415807 | 89472841 | + | gene9107 | TMPRSS9 | protein_coding |
| 464966 | 7 | 66987811 | 67005233 | - | gene8736 | PWWP3A | protein_coding |
| 1040202 | 18 | 3404269 | 3421248 | - | gene20165 | RDH13 | protein_coding |
| 114802 | 2 | 89017533 | 89089426 | + | gene1869 | AOX1 | protein_coding |
| 1435571 | 26 | 32813595 | 32820300 | + | gene28285 | EXOSC1 | protein_coding |
| 534248 | 8 | 24934728 | 24936524 | - | gene10139 | RRAGA | protein_coding |
| 622327 | 9 | 103663668 | 103835624 | - | gene11753 | WDR27 | protein_coding |
| 955036 | 16 | 32974926 | 33226324 | - | gene18528 | SDCCAG8 | protein_coding |
| 1094312 | 18 | 57241472 | 57254867 | + | gene21517 | SDR42E1 | protein_coding |
| 924315 | 15 | 54531234 | 54537045 | - | gene17932 | PDZD3 | protein_coding |
| 297599 | 4 | 106919355 | 107015240 | - | gene5432 | ASB4 | protein_coding |
| 1262905 | 22 | 16935381 | 16969582 | - | gene24979 | ASB14 | protein_coding |
| 151339 | 2 | 128584619 | 128588677 | - | gene2418 | PNRC2 | protein_coding |
| 461623 | 7 | 64386108 | 64427982 | - | gene8671 | PCBD2 | protein_coding |
| 30879 | 1 | 81417061 | 81426699 | + | gene605 | TMEM41A | protein_coding |
| 715140 | 11 | 37618063 | 37652642 | - | gene13553 | CLHC1 | protein_coding |
| 686036 | 10 | 97990774 | 98004250 | + | gene13095 | KIAA1191 | protein_coding |
| 933440 | 15 | 66216579 | 66306449 | + | gene18089 | C15H11orf65 | protein_coding |
| 903540 | 15 | 19659149 | 19671382 | + | gene17200 | C15H11orf91 | protein_coding |
| 867855 | 14 | 15947327 | 15951767 | - | gene16275 | TRMT12 | protein_coding |
| 796541 | 12 | 86194012 | 86212119 | - | gene14981 | GRTP1 | protein_coding |
| 673173 | 10 | 70354495 | 70415128 | + | gene12842 | MTHFD1 | protein_coding |
| 1261020 | 22 | 13632608 | 13724811 | - | gene24955 | CHDH | protein_coding |
| 988424 | 16 | 79255366 | 79343117 | + | gene19136 | SYT2 | protein_coding |
| 715294 | 11 | 37655989 | 37678560 | - | gene13556 | MTIF2 | protein_coding |
| 673382 | 10 | 70495529 | 70522939 | + | gene12847 | PPP1R36 | protein_coding |
| 476864 | 7 | 88762978 | 88872530 | + | gene9080 | RAD50 | protein_coding |
| 991946 | 17 | 274033 | 304701 | + | gene19181 | GNB1L | protein_coding |
| 1376451 | 25 | 14781525 | 14792832 | + | gene27302 | PHKG1 | protein_coding |
| 610115 | 9 | 81552485 | 81583657 | + | gene11537 | STX11 | protein_coding |
| 609990 | 9 | 81336502 | 81370346 | + | gene11533 | ZC2HC1B | protein_coding |
| 1134230 | 19 | 35598861 | 35610578 | + | gene22376 | ATPAF2 | protein_coding |
| 352262 | 5 | 63342345 | 63367655 | - | gene6612 | TIMELESS | protein_coding |
| 59925 | 1 | 148654179 | 148666971 | - | gene1230 | PIGP | protein_coding |
| 1143711 | 19 | 41318183 | 41331414 | - | gene22567 | GSDMB | protein_coding |
| 1483193 | 28 | 43900463 | 44015003 | - | gene29283 | PARG | protein_coding |
| 338443 | 5 | 45227065 | 45237328 | + | gene6261 | TXN2 | protein_coding |
| 240170 | 3 | 110824386 | 110827901 | + | gene4249 | TMEM35B | protein_coding |
| 1202603 | 21 | 21348836 | 21357354 | - | gene23939 | ARPIN | protein_coding |
| 566545 | 8 | 83898381 | 83924837 | - | gene10665 | NOL8 | protein_coding |
| 965510 | 16 | 46212692 | 46230050 | + | gene18708 | NOL9 | protein_coding |
| 484126 | 7 | 91997900 | 92002817 | + | gene9205 | HSD11B1L | protein_coding |
| 682890 | 10 | 82420073 | 82447005 | - | gene13017 | VIPAS39 | protein_coding |
| 991824 | 17 | 239530 | 267584 | + | gene19180 | TXNRD2 | protein_coding |
| 142531 | 2 | 121288963 | 121308413 | - | gene2243 | TMEM39B | protein_coding |
| 1146750 | 19 | 43410109 | 43414593 | - | gene22686 | GHDC | protein_coding |
| 456288 | 7 | 58982602 | 58989642 | - | gene8573 | HARS2 | protein_coding |
| 1354997 | 24 | 56982933 | 56999714 | - | gene26907 | NARS | protein_coding |
| 964685 | 16 | 44949636 | 44963454 | - | gene18698 | PER3 | protein_coding |
| 1588383 | NW_020867367.1 | 13254 | 59059 | - | gene32097 | RAD50 | protein_coding |
| 1071501 | 18 | 20167644 | 20168798 | + | gene21000 | CALM2 | protein_coding |
| 216181 | 3 | 86182305 | 86213829 | + | gene3852 | CYP2J2 | protein_coding |
| 1519002 | 29 | 31963035 | 32098012 | - | gene30067 | GDPD4 | protein_coding |
| 1043881 | 18 | 5362095 | 5384992 | + | gene20276 | ZNF271P | protein_coding |
| 33720 | 1 | 82754950 | 82762873 | - | gene637 | HTR3E | protein_coding |
| 201729 | 3 | 54339189 | 54422506 | + | gene3616 | GBP2 | protein_coding |
| 641597 | 10 | 20947083 | 20948014 | - | gene12260 | TTC9C | protein_coding |
|  | 4 | 105294633 | 105296692 | + | gene5417 | SEC24A | protein_coding |
| 1590201 | NW_020867550.1 | 2017 | 36276 | - | gene32234 | SLC25A20 | protein_coding |
| 1157485 | 19 | 49310347 | 49332881 | - | gene22865 | ICAM2 | protein_coding |
| 132446 | 2 | 108813436 | 108815859 | + | gene2087 | PNRC2 | protein_coding |
| 914284 | 15 | 38150057 | 38193953 | + | gene17723 | GVINP1 | protein_coding |
| 211573 | 3 | 76966711 | 76969039 | - | gene3768 | TTC9C | protein_coding |
| 1591971 | NW_020867680.1 | 20086 | 21229 | - | gene32403 | IFITM3 | protein_coding |
| 1587341 | NW_020867178.1 | 2844 | 29359 | + | gene32020 | ABHD6 | protein_coding |
| 1145907 | 19 | 43052428 | 43057048 | - | gene22665 | SNURF | protein_coding |
| 201648 | 3 | 54248316 | 54264606 | + | gene3614 | GBP5 | protein_coding |
| 558107 | 8 | 71338966 | 71340528 | - | gene10528 | RRAGA | protein_coding |
| 327215 | 5 | 21611105 | 21646177 | + | gene6061 | RBP1 | protein_coding |
| 1030419 | 17 | 43256608 | 43257049 | - | gene19803 | COMMD6 | protein_coding |
| 1588788 | NW_020867410.1 | 871 | 12624 | + | gene32119 | CDC40 | protein_coding |
| 1044514 | 18 | 6474939 | 6487782 | + | gene20320 | ZNF415 | protein_coding |
| 1057486 | 18 | 13679973 | 13703160 | - | gene20692 | ZNF226 | protein_coding |
| 461329 | 7 | 63414689 | 63434336 | - | gene8658 | FBXL21P | protein_coding |
| 1588203 | NW_020867342.1 | 3924 | 36411 | - | gene32082 | CYB5R4 | protein_coding |
| 1588430 | NW_020867375.1 | 32871 | 46352 | + | gene32101 | KIAA1191 | protein_coding |
| 1096417 | 19 | 7379536 | 7381828 | - | gene21649 | THAP12 | protein_coding |
| 1157446 | 19 | 49276863 | 49306580 | - | gene22864 | ICAM2 | protein_coding |
| 240178 | 3 | 110828883 | 110832759 | + | gene4250 | PTAFR | protein_coding |
| 157064 | 2 | 133055000 | 133066811 | - | gene2506 | AKR7A3 | protein_coding |
| 1587501 | NW_020867197.1 | 14865 | 67111 | + | gene32032 | ZRANB1 | protein_coding |
| 1594998 | NW_020868201.1 | 53 | 14411 | + | gene32763 | PM20D2 | protein_coding |
| 1587427 | NW_020867186.1 | 447 | 17112 | + | gene32027 | GMPS | protein_coding |
| 1588893 | NW_020867418.1 | 27979 | 49013 | + | gene32126 | LRRCC1 | protein_coding |
| 1587824 | NW_020867255.1 | 6924 | 30512 | - | gene32055 | TTC3 | protein_coding |

Supplementary Table 2: Significant tissue specific GO terms which are based on tissue specific DEGs between Bt and Bi. N: total number of DEGs in a given GO term. Up: number of DEGs with higher expression in Angus for given GO term. Down: number of DEGs higher expression in Brahman for a given GO term. P.up: P-value for DEGs with increased expression. P.down: P-value for DEGs with reduced expression.

| Liver | **Term** | **Ontology** | **N** | **Up** | **Down** | **P.Up** | **P.Down** |
| --- | --- | --- | --- | --- | --- | --- | --- |
| **GO:0043167** | ion binding | MF | 16 | 3 | 13 | 0.99181787 | 0.03493364 |
| **GO:0072359** | circulatory system development | BP | 5 | 0 | 5 | 1 | 0.05826632 |
| **GO:0071704** | organic substance metabolic process | BP | 20 | 5 | 15 | 0.9764922 | 0.06922876 |
| **GO:0043169** | cation binding | MF | 14 | 3 | 11 | 0.97895504 | 0.07749002 |
| **GO:0046872** | metal ion binding | MF | 14 | 3 | 11 | 0.97895504 | 0.07749002 |
| **GO:0044238** | primary metabolic process | BP | 19 | 5 | 14 | 0.96506196 | 0.09672046 |
|  |  |  |  |  |  |  |  |
| Muscle | **Term** | **Ontology** | **N** | **Up** | **Down** | **P.Up** | **P.Down** |
| **GO:0071944** | cell periphery | CC | 12 | 11 | 1 | 0.0396676 | 0.99520502 |
| **GO:0016020** | membrane | CC | 19 | 16 | 3 | 0.05210276 | 0.98691193 |
| **GO:0016021** | integral component of membrane | CC | 11 | 10 | 1 | 0.058275 | 0.99229896 |
| **GO:0031224** | intrinsic component of membrane | CC | 11 | 10 | 1 | 0.058275 | 0.99229896 |
| **GO:0005886** | plasma membrane | CC | 11 | 10 | 1 | 0.058275 | 0.99229896 |
| **GO:0005623** | cell | CC | 22 | 18 | 4 | 0.06223357 | 0.98147157 |
| **GO:0044464** | cell part | CC | 22 | 18 | 4 | 0.06223357 | 0.98147157 |
| **GO:0005576** | extracellular region | CC | 8 | 3 | 5 | 0.98131244 | 0.08927174 |
| **GO:0048584** | positive regulation of response to stimulus | BP | 4 | 1 | 3 | 0.98067283 | 0.09494253 |
|  |  |  |  |  |  |  |  |
| Brain | **Term** | **Ontology** | **N** | **Up** | **Down** | **P.Up** | **P.Down** |
| **GO:0016043** | cellular component organization | BP | 5 | 0 | 5 | 1 | 0.0149365 |
| **GO:0071840** | cellular component organization or biogenesis | BP | 5 | 0 | 5 | 1 | 0.0149365 |
| **GO:1901363** | heterocyclic compound binding | MF | 5 | 0 | 5 | 1 | 0.0149365 |
| **GO:0097159** | organic cyclic compound binding | MF | 5 | 0 | 5 | 1 | 0.0149365 |
| **GO:0022607** | cellular component assembly | BP | 4 | 0 | 4 | 1 | 0.03561781 |
| **GO:0044085** | cellular component biogenesis | BP | 4 | 0 | 4 | 1 | 0.03561781 |
| **GO:0005576** | extracellular region | CC | 4 | 4 | 0 | 0.09127434 | 1 |
| **GO:0044421** | extracellular region part | CC | 4 | 4 | 0 | 0.09127434 | 1 |
| **GO:0005488** | binding | MF | 16 | 6 | 10 | 0.96965011 | 0.09279014 |
|  |  |  |  |  |  |  |  |
| Lung | **Term** | **Ontology** | **N** | **Up** | **Down** | **P.Up** | **P.Down** |
| **GO:0070887** | cellular response to chemical stimulus | BP | 5 | 0 | 5 | 1 | 0.01568686 |
| **GO:0042221** | response to chemical | BP | 5 | 0 | 5 | 1 | 0.01568686 |
| **GO:0071495** | cellular response to endogenous stimulus | BP | 4 | 0 | 4 | 1 | 0.03669129 |
| **GO:0071310** | cellular response to organic substance | BP | 4 | 0 | 4 | 1 | 0.03669129 |
| **GO:0009719** | response to endogenous stimulus | BP | 4 | 0 | 4 | 1 | 0.03669129 |
| **GO:0010033** | response to organic substance | BP | 4 | 0 | 4 | 1 | 0.03669129 |
| **GO:0050789** | regulation of biological process | BP | 13 | 4 | 9 | 0.98595018 | 0.05491781 |
| **GO:0065007** | biological regulation | BP | 15 | 5 | 10 | 0.98287617 | 0.05922439 |
| **GO:0065009** | regulation of molecular function | BP | 6 | 1 | 5 | 0.99335885 | 0.06091539 |
| **GO:0009987** | cellular process | BP | 17 | 6 | 11 | 0.98031554 | 0.06220965 |
|  |  |  |  |  |  |  |  |
| Placenta | **Term** | **Ontology** | **N** | **Up** | **Down** | **P.Up** | **P.Down** |
| **GO:0005737** | cytoplasm | CC | 25 | 17 | 8 | 0.00874592 | 0.99752865 |
| **GO:0043168** | anion binding | MF | 5 | 5 | 0 | 0.01511471 | 1 |
| **GO:0036094** | small molecule binding | MF | 5 | 5 | 0 | 0.01511471 | 1 |
| **GO:0005622** | intracellular | CC | 28 | 18 | 10 | 0.01647333 | 0.9944948 |
| **GO:0044424** | intracellular part | CC | 28 | 18 | 10 | 0.01647333 | 0.9944948 |
| **GO:0044444** | cytoplasmic part | CC | 14 | 10 | 4 | 0.03028848 | 0.99281735 |
| **GO:0005515** | protein binding | MF | 14 | 10 | 4 | 0.03028848 | 0.99281735 |
| **GO:0097367** | carbohydrate derivative binding | MF | 4 | 4 | 0 | 0.03537484 | 1 |
| **GO:0097458** | neuron part | CC | 4 | 4 | 0 | 0.03537484 | 1 |
| **GO:1901265** | nucleoside phosphate binding | MF | 4 | 4 | 0 | 0.03537484 | 1 |
| **GO:0000166** | nucleotide binding | MF | 4 | 4 | 0 | 0.03537484 | 1 |
| **GO:0017076** | purine nucleotide binding | MF | 4 | 4 | 0 | 0.03537484 | 1 |
| **GO:0035639** | purine ribonucleoside triphosphate binding | MF | 4 | 4 | 0 | 0.03537484 | 1 |
| **GO:0032555** | purine ribonucleotide binding | MF | 4 | 4 | 0 | 0.03537484 | 1 |
| **GO:0032553** | ribonucleotide binding | MF | 4 | 4 | 0 | 0.03537484 | 1 |
| **GO:0065008** | regulation of biological quality | BP | 9 | 7 | 2 | 0.03920414 | 0.99356306 |
| **GO:0005623** | cell | CC | 34 | 20 | 14 | 0.04169907 | 0.98237776 |
| **GO:0044464** | cell part | CC | 34 | 20 | 14 | 0.04169907 | 0.98237776 |
| **GO:0003008** | system process | BP | 6 | 5 | 1 | 0.05859537 | 0.99358143 |
| **GO:0003824** | catalytic activity | MF | 17 | 11 | 6 | 0.06012427 | 0.98052631 |
